# Knockdown resistance mutations distribution and characteristics of *Aedes albopictus* field populations within eleven dengue local epidemic provinces in China

**DOI:** 10.3389/fcimb.2022.981702

**Published:** 2023-02-09

**Authors:** Chunchun Zhao, Xinxin Zhou, Chuizhao Xue, Xinchang Lun, Wenyu Li, Xiaobo Liu, Haixia Wu, Xiuping Song, Jun Wang, Qiyong Liu, Fengxia Meng

**Affiliations:** ^1^ State Key Laboratory of Infectious Disease Prevention and Control, World Health Organization (WHO) Collaborating Centre for Vector Surveillance and Management, Chinese Center for Disease Control and Prevention, National Institute for Communicable Disease Control and Prevention, Beijing, China; ^2^ Beijing Daxing District Center for Disease Control and Prevention, Genaral Office, Beijing, China; ^3^ National Institute of Parasitic Diseases, Chinese Center for Disease Control and Prevention (Chinese Center for Tropical Diseases Research), National Health Committee (NHC) Key Laboratory of Parasite and Vector Biology, World Health Organization (WHO) Collaborating Centre for Tropical Diseases, National Center for International Research on Tropical Diseases, Shanghai, China

**Keywords:** *Aedes albopictus*, resistance, *kdr* gene, pyrethroid insecticides, correlation analysis

## Abstract

**Background:**

*Aedes albopictus*, commonly known as the tiger mosquito, has attracted global attention because its bite can transmit several viruses, such as dengue virus. With the absence of an effective therapy and vaccine, mosquito control is the sole method for dengue fever control. However, *Ae. albopictus* has developed resistance to most insecticides, especially pyrethroids. Many scholars have conducted thorough research for the target-site of pyrethroids. The main target-site is the voltage-gated sodium channel gene (*VGSC*) whose mutation causes knockdown resistance (*kdr*). The spatial distribution of three locus *kdr* mutations in *Ae. albopictus* has not been comprehensively analyzed nationwide in China. In addition, the relationship between the frequency of *kdr* mutations and dengue fever has not yet been explored.

**Methods:**

A total of 2,241 *Ae. albopictus* samples from 49 populations from 11 provinces of mainland China were collected in 2020 and analyzed for mutations in the *VGSC* gene. DNAstar 7.1. Seqman and Mega-X were used to compare the sequences and read the peak map to confirm the genotypes and alleles of each mutation. ArcGIS 10.6 software was used to make interpolation and extract meteorological data of collection sites and to conduct spatial autocorrelation analysis. R 4.1.2 software was used to conduct a chi-square test for *kdr* mutations and dengue area and to analyze the correlation between meteorological factors and *kdr* mutations.

**Results:**

The overall frequencies of mutant alleles at 1016G, 1532T, and 1534S/C/L were 13.19%, 4.89%, and 46.90%, respectively. Mutations at the three loci were found at 89.80% (44/49), 44.90% (22/49), and 97.96% (48/49) of the field populations. At each of the loci V1016 and I1532, only one allele was detected, which was GGA(G) and ACC(T), respectively. Five mutant alleles were found at codon 1534: TCC/S (33.49%), TGC/C (11.96%), TTG/L (0.60%), CTC/L (0.49%), and TTA/L (0.58%). In total, 31 triple-locus genotype combinations were found, and the single locus mutation was the most common. We also found firstly triple-locus mutant individuals, whose genotypes were V/G+I/T+F/S and V/G+I/T+S/S. The 1016 and 1532 mutation rates were significantly negatively related to the annual average temperature (AAT), but the 1534 mutation rate was significantly positively related to AAT. The 1532 mutation rate was significantly positively related to the 1016 mutation rate but negatively related to the 1534 mutation rate. A relationship was observed between the 1534 codon mutation rate and dengue epidemic areas in this study. Furthermore, spatial autocorrelation analysis results showed that the mutation rates of different codons in different geographical areas had spatial aggregation and positive spatial correlation.

**Conclusion:**

This study showed that the multiple *kdr* mutations at codon 1016, 1532 and 1534 of *Ae. albopictus* were found in most areas of China. Two novel triple-locus genotype combinations, V/G+I/T+F/S and V/G+I/T+S/S, were detected in this study. In addition, the relationship between mosquito resistance and dengue fever outbreak should be further explored, especially considering the insecticide-usage history in different areas. The characteristic of spatial aggregation of *VGSC* gene mutation rates reminds us to notice the gene exchange and similarity of insecticide usage in the adjacent areas. The use of pyrethroids should be restricted to delay resistance development. New-type insecticides should be developed to adjust the changes in the resistance spectrum. Our study provides abundant data on the *Ae. albopictus kdr* gene mutation in China; these findings will be useful for the correlation analysis of molecular mechanism of insecticide resistance.

## Introduction

As an important vector, *Ae. albopictus* can transmit several viruses, such as dengue virus, chikungunya virus, yellow fever virus and Zika virus (Smith et al., 2016). *Ae. albopictus* ranks second only to *Ae. aegypti* in importance to humans as a vector of dengue virus (Knudsen, 1995), which affect millions in the tropical and subtropical regions of the world (Lee and Rohani, 2005). Dengue virus is a big threat in China (Yue et al., 2019). As of 2019, there have been local outbreaks of dengue fever recorded in 13 provinces in the mainland of China (Lun et al., 2022).

*Ae. albopictus* has been described as one of the 100 worst invasive species in the world (Goubert et al., 2016) and is one of the most common mosquito species in China; it has been found in nearly one third of China (Wu et al., 2021) and is the major vector of dengue virus in China (Wu et al., 2014). Thus, there is a significant risk that dengue fever will be transmitted throughout China.

As a result of the absence of specific antiviral drugs or a vaccine, vector control is the sole viable method for reducing the disease burden (Almeida et al., 2005; Wilder-Smith et al., 2010). Owing to their efficacy, insecticides are predominantly used to decrease mosquito density and reduce the risk of dengue transmission (Ishak et al., 2015; Yiguan et al., 2017). Pyrethroids are still the most used adulticides for *Aedes* mosquitoes because of their rapid activity in insects and low mammalian toxicity (Moyes et al., 2017). However, long-term and mass application of pyrethroid insecticides has caused the serious problem of insecticide resistance (Gao et al., 2018; Li et al., 2021), which is an enormous challenge for the prevention and control of vector-borne diseases.

Insecticide resistance can be related to changes in the sequence of the target protein that induce insensitivity to the insecticide (target-site resistance), and/or to the upregulation of detoxification enzymes (Marcombe et al., 2014). The main target-site resistance mechanisms known in mosquitoes involve amino acid substitutions in the voltage-gated sodium channel (*VGSC*) that cause resistance to pyrethroid insecticides, known as knockdown resistance (*kdr*) (Brengues et al., 2003). One of the structure for *VGSC* is α-Subunit which contains four homologous domains (I-IV) (Loughney et al., 1989; Rinkevich et al., 2013). The *kdr* mutation, F1534C, in *Ae. albopictus* was first detected in Singapore in 2009 (Kasai et al., 2011); it is one of the most widespread and analyzed mechanisms causing resistance to DDT and pyrethroids. More *kdr* mutations have since been found in several other countries (Xu et al., 2016; Kasai et al., 2019; Saha et al., 2019). Currently, there are three locus mutations in *VGSC* at codon 1016 of domain II and codons 1532 and 1534 of domain III that are correlated with pyrethroid insecticide resistance of *Ae. albopictus* (Auteri et al., 2018). Xu et al. (Xu et al., 2016) and Wu et al. (Wu et al., 2021) showed a positive association between the F1534 mutation and pyrethroid resistance, but possibly a negative correlation with the I1532 mutation. Kasai et al. (Kasai et al., 2019) first detected V1016G in *Ae. albopictus* collected from Italy and Vietnam and then showed that it was correlated with a high level of insecticide resistance. Zhou et al. (Zhou et al., 2019) reported the novel V1016G mutation in Beijing, China. Previous studies state that temperature is the most important ecological factor affecting the physiology of *Aedes* species, in addition to the gonotrophic cycle length and the survival rate of adults (Brady et al., 2014). Chen et al. (Chen et al., 2021) analyzed the correlation between annual average temperature, and *kdr* mutations, indicating that temperature possibly affects insecticide resistance. In addition, it has been reported that multiple loci mutations in the *VGSC* gene can simultaneously increase the level of resistance (Plernsub et al., 2016).

With globalization, the trade between China and other countries is increasing gradually. The spread of *kdr* mutations of *Ae. albopictus* is linked to the geographical spread of mosquito eggs in container shipments of tires and plants (Sprenger, 1987). In the present study, we analyzed the *kdr* mutations found in 49 field populations from 11 provinces of China where there were local outbreaks of dengue fever. We also compared the mutation rate in different areas and further explored the factors that affect resistance.

## Methods and materials

### Sample collection

*Ae. albopictus* samples were collected from 49 sites located in 11 provinces of mainland China in which there have been local outbreaks of dengue fever as of 2019. The provinces were Henan, Guangxi, Hunan, Chongqing, Hubei, Guangdong, Shandong, Yunnan, Jiangxi, Zhejiang, and Fujian. Information regarding the collection sites is described in **Table 1**.

**Table 1:** Brief information for *Ae. albopictus* collection in China.

Province	City/District	Code	Environment	Province	City/District	Code	Environment
Henan	Luoyang	HALY	Waste station	Hubei	Yichang	HBYC	Residential area
	Xuchang	HAXC	Residential area		Jingzhou	HBJZ	Garden
	Puyang	HAPY	Residential area		Xiangyang	HBXY	Residential area
	Jiaozuo	HAJZ	Discarded tire pile	Guangdong	Foshan	GDFS	Residential area
	Nanyang	HANY	Construction site		Guangzhou	GDGZ	Residential area
Guangxi	Nanning	GXNN	Residential area		Jiangmen	GDJM	Residential area
	Wuzhou	GXWZ	Water container		Shenzhen	GDSZ	Residential area
	Beihai	GXBH	Residential area		Shaoguan	GDSG	Residential area
	Qingzhou	GXQZ	Waste bucket		Maoming	GDMM	Residential area
	Fangchenggang	GXFCG	Water container	Shandong	Jining	SDJX	Residential area
	Baise	GXBS	Discarded tire pile		Weifang	SDWF	Residential area
Hunan	Hengyang	HNHY	Residential area		Zibo	SDZB	Residential area
	Loudi	HNLD	Flower market		Jinan	SDLC	Garden
	Huaihua	HNHH	Residential area	Yunnan	Puer	YNLC	Residential area
	Zhuzhou	HNZZ	Flower market		Honghe	YNHK	Residential area
	Xiangtan	HNXT	Flower market		Dehong	YNMS	Residential area
Chongqing	Yubei	CQYB	Residential area	Jiangxi	Nanchang	JXNCX	Residential area
	Wanzhou	CQWZ	Residential area		Ganzhou	JXGZ	Residential area
	Shapingba	CQSPB	School		Xihu	JXXHQ	Hospital
	Qianjiang	CQQJ	Hospital	Zhejiang	Quzhou	ZJQZ	Residential area
	Dazu	CQDZ	Residential area		Jinhua	ZJYW	Residential area
	Dianjiang	CQDJ	Discarded tire pile		Wenzhou	ZJWZ	Residential area
Hubei	Huangzhou	HBHZ	Residential area	Fujian	Huli	FJHL	Garden
	Wuxue	HBWX	Residential area		Xiangan	FJXA	Hospital
	Wuhan	HBWH	Residential area		–	–	–

Adult mosquitoes were captured by the human-baited double net trap (Tangena et al., 2015) from 15:00 to 18:00 h, during which *Aedes* have peak activity, and larva were collected by standard dipping method (Farjana et al., 2015). The samples were provided by local Centers for Disease Control and Prevention (CDCs). Larvae were identified by a PCR method, as established by Higa et al. (Higa et al., 2010), and adults by morphology.

### DNA extraction, amplification, and sequence analysis of *VGSC* fragments

Genomic DNA from each alcohol-preserved adults and larvae was extracted using Micro Tissue Genomic DNA Extraction Kit (BioTeke, Wuxi, China) and DNA/RNA Extraction-32 system (BioTeke, Wuxi, China) according to the manufacturer’s instructions.

Primers (Kasai et al., 2019) aegSCF20 (5′-GAC AAT GTG GAT CGC TTC CC-3′) and aegSCR21 (5′-GCA ATC TGG CTT GTT AAC TTG-3′) were used to amplify the partial sequence of domain II covering codon 1016, and primers aegSCF7 (5′-GAG AAC TCG CCG ATG AAC TT-3′) and aegSCR7 (5′-GAC GAC GAA ATC GAA CAG GT-3′) were used to amplify the fragment of domain III containing codons 1532 and 1534.

For domain II, the reaction was performed in a final volume of 25 μL, comprising 12.5 μL of 2× Easy TaqTM PCR SuperMix (+dye) (TransGen Biotech, Beijing, China), 1 μL of 10 μM aegSCF20, 1μL of 10 μM aegSCR21, 2 μL of DNA template, and ddH_2_O to make up to 25 μL. The reaction procedure was as follows: 94°C for 3 min, followed by 35 cycles of 94°C for 30 s, 58°C for 30 s, and 72°C for 30 s, with a final extension at 72°C for 10 min. The PCR products were gel-purified and directly sequenced by TsingKe Co. Ltd (Beijing, China) in a single direction using the reverse primer aegSCR22 (5′-TTC ACG AAC TTG AGC GCG TTG-3′).

For domain III, the reaction was done in a final volume of 25 μL, comprising 12.5 μL of 2× Easy TaqTM PCR SuperMix (+dye) (TransGen Biotech, Beijing, China), 1 μL of 10 μM aegSCF7, 1 μL of 10 μM aegSCR7, 2 μl of DNA template, and ddH_2_O to make up to 25 μL. The reaction procedure was as follows: 94°C for 3 min, followed by 35 cycles of 94°C for 30 s, 56°C for 30 s, and 72°C for 30 s, with a final extension at 72°C for 10 min. The PCR products were gel-purified and directly sequenced by TsingKe Co. Ltd (Beijing, China) in a single direction using the reverse primer aegSCR8 (5′-TAG CTT TCA GCG GCT TCT TC-3′).

Sequence analysis: Seqman of DNAStar 7.1. and Mega-X software were used to compare the sequences and read the peak map to confirm the genotype and allele of each mutation site. If the chromatograms show there are two peaks of equal height at one site, the sample is regarded as heterozygote individual. The alleles, genotypes, frequencies, and combined mutations of the *VGSC* gene in different populations of *Ae. albopictus* were calculated and analyzed using WPS Office 2021 software (Kingsoft, Beijing, China).

### Annual average temperature and average annual rainfall of sample collection sites

We collected daily temperature and rainfall data from January 2011 to December 2020 for the 11 study provinces from National Centers for Environmental Information (NCEI, https://www.ncei.noaa.gov/). R 4.1.2 software was used to calculate the annual average temperature (AAT) and average annual rainfall (AAR). ArcGIS 10.6 software was used to conduct spatial interpolation by an inverse distance weighted (IDW) method and the data on study sites were extracted from IDW results.

### Statistical analysis

Comparison of mutation rates between dengue epidemic areas (DEAs) and non-dengue epidemic areas (NDEAs) was performed using chi-square test and fisher’s exact test and *P*<0.05 indicates there are significant difference among groups. The number of wild alleles was obtained by double number of wild homozygotes and the number of wild-type-mutant heterozygote. The number of mutant alleles was from which double number of samples deducted the number of wild alleles. Relationship between different factors was performed using correlation analysis. The chi-square test, fisher’s exact test and correlation analysis were conducted using R 4.1.2 software. Global and local spatial autocorrelation analysis was conducted using ArcGIS 10.6 software. Moran’s *I* index was used for global spatial autocorrelation analysis, and Anselin Local Moran’s *I* index and Getis-ord Gi* were used for local spatial autocorrelation analysis (Lun et al., 2022).

## Results

### Mosquito samples and collection sites

**Figure 1:**
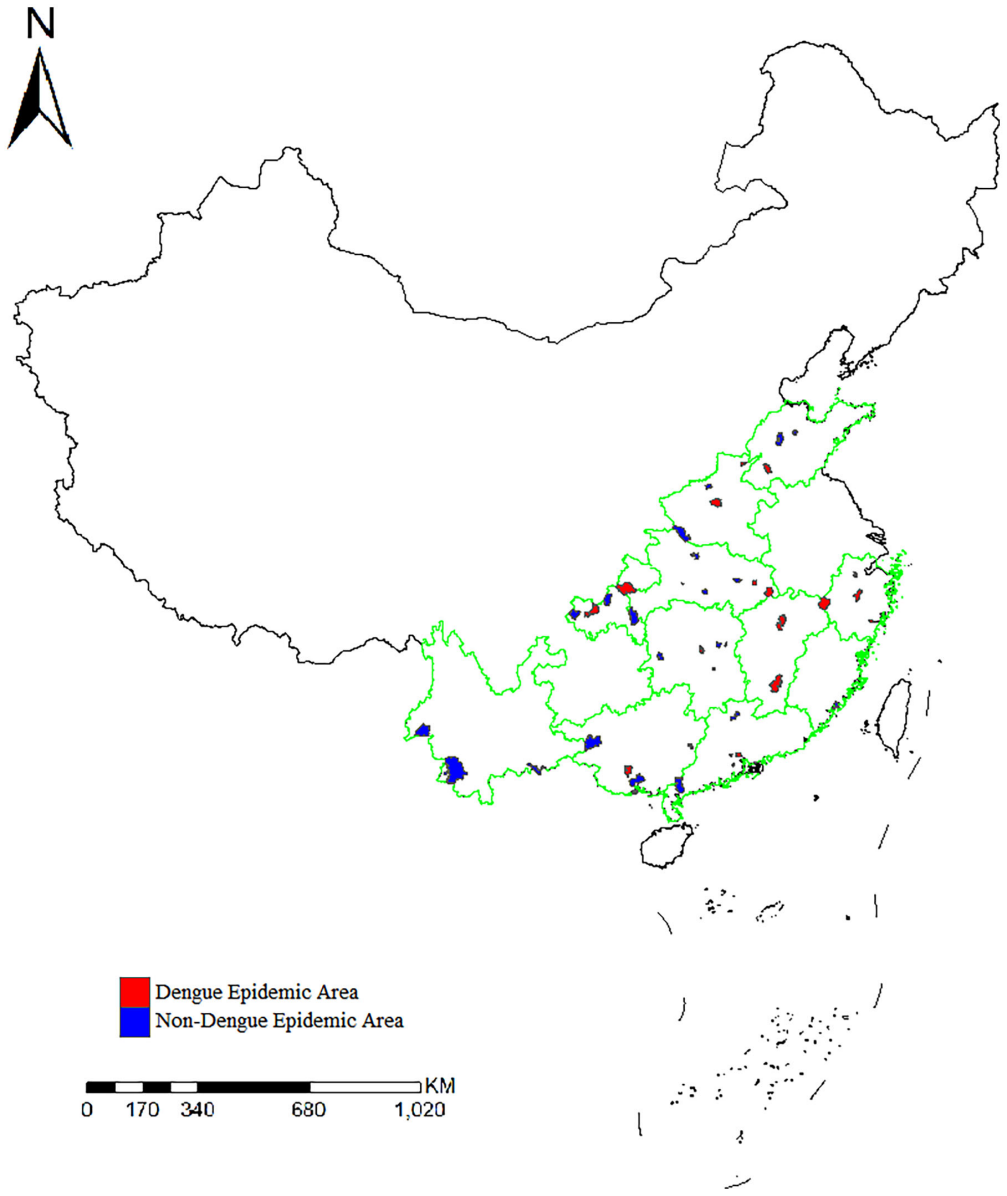
Distribution of the sample collection sites and location of dengue fever outbreak areas in 11 provinces.

A total of 2241 *Ae. albopictus* adults and larvae were collected from 49 sites in 11 provinces in mainland China in 2020. The number of samples collected at each site ranged from 29 to 87. The number of sites in each province was two to six. There had been local outbreaks of dengue fever as of 2019 at 24 sample collection sites (**Figure 1**).

### V1016 mutation rate at codon and distribution in the field populations

**Figure 2:**
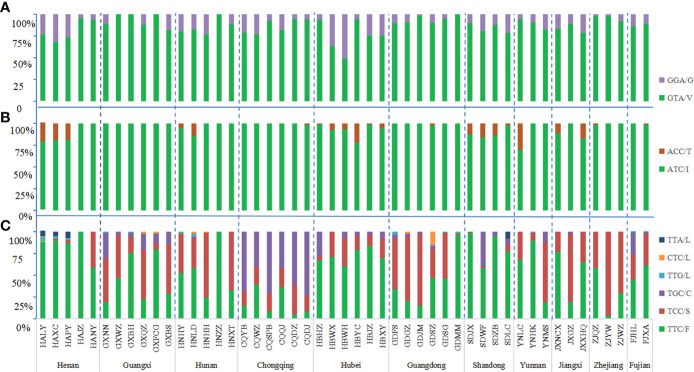
Histogram of allele frequency at codons 1016 **(A)**, 1532 **(B)** and 1534 **(C)** of the *VGSC* in *Ae. albopictus* populations. For detailed information, see **Table S1**. The dotted line separates the sites by each province. The horizontal axis indicates the populations (see **Table 1** and **Figure 1** for detail), while the vertical axis denotes the allele frequency.

**Figure 3:**
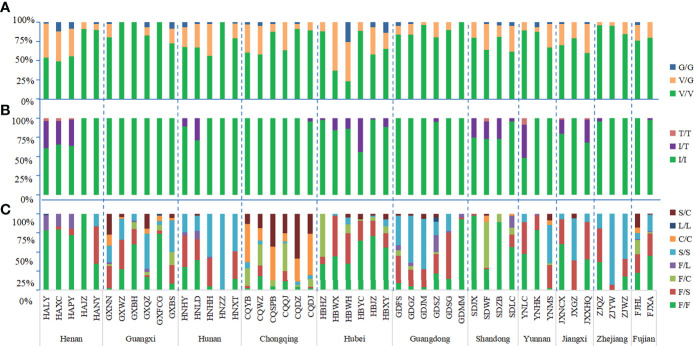
Histogram of genotype frequency at codons 1016 **(A)**, 1532 **(B)** and 1534 **(C)** of the the VGSC in *Ae. albopictus* populations. The dotted line separates the sites by each province. The horizontal axis indicates the populations (see **Table 1** and **Figure 1** for detail), while the vertical axis denotes the genotype frequency.

The wild-type allele at the 1016 codon was GTA(V) and one mutant allele GGA(G) were found. The mutation rate at 1016 codon was 13.19%. Three genotypes were detected, wild-type genotype V/V (76.62%), wild-type-mutant heterozygote V/G (20.39%), and mutant genotype G/G (2.99%) (**Figures 2A**, **3A**; **Table S1**).

The mutant allele GGA(G) was detected in 44 field populations in which the highest frequency was 51.16% in HBWH, followed by HBWX of Hubei province. The other 43 populations had frequencies less than 50%, with the lowest frequency of 1.72% in GDJM of Guangdong province. The mutant homozygous 1016 G/G was detected in 28 field populations and the highest frequency was 25.38% which also was in HBWH. The lowest frequency was 1.79% in HALY of Henan province. The wild-type-mutant heterozygote 1016 V/G was found in 44 populations, with a frequency ranging from 3.45% to 63.16% (**Table S2**).

### I1532 mutation rate at codon and distribution in the field populations

In addition to the wild-type allele ATC(I), only one mutant allele ACC(T) was found at codon 1532 and the mutation rate was 4.89%. Three genotypes were found: wild-type genotype I/I (90.81%), wild-type-mutant heterozygote I/T (8.61%), and mutant genotype T/T (0.58%) (**Figures 2B**, **3B**; **Table S1**).

The mutant allele ACC(T) was detected in 22 field populations with a mutation frequency ranging from 1.11% to 30.43%. The mutant homozygous 1532 T/T was detected in seven populations: YNLC (8.70%) of Yunnan province, SDWF (4.26%) of Shandong province, HAXC (4.08%), HALY (3.57%), and HAPY (2.13%) of Henan province, JXNCX (2.33%), and JXXHQ (2.13%) of Jiangxi province (**Table S2**). No mutation was found in the 1532 codon in the sample collection sites of Guangxi province.

### F1534 mutation rate at codon and distribution in the field populations

The wild-type codon at the 1534 codon was TTC(F). The overall average frequency of the mutant allele F1534 across all collection sites was 46.99%, which was the codon with the highest mutation frequency and highest prevalence in the *VGSC* gene of *Ae. albopictus*. Five mutant alleles were detected: TCC(S) 33.36%, TGC(C) 11.96%, TTG(L) 0.60%, CTC(L) 0.49%, and TTA(L) 0.58%. A total of eight genotypes were found in this study, the wild-type genotype F/F (37.26%); three wild-type-mutant heterozygotes F/S (21.37%), F/C (7.14%), and F/L (2.99%); three mutant homozygotes S/S (19.46%), C/C (5.18%), and L/L (0.18%); and one mutant-type-mutant heterozygote S/C (6.48%), indicating that 62.74% of *Ae. albopictus* carried mutations at the 1534 codon of the *VGSC* gene (**Figures 2C**, **3C**; **Table S1**).

Of the 49 field populations studied, 48 had F1534 mutations. The only one population without the mutations was HAJZ of Henan province. The highest frequencies were 100% in the following populations: HNZZ of Hunan province, JXGZ of Jiangxi province, and ZJYW of Zhejiang province; followed by the populations collected at CQDJ (97.83%), CQYB (97.37%), CQDZ (95.65%), CQSPB (95%) of Chongqing; GXNN (97.56%), GXBS (91.67%) of Guangxi; GDJM (96.55%), GDGZ (95.45%), GDFS (90.91%) of Guangdong; and HNHH (95.35%) of Hunan Province. There were 20 populations with frequencies between 50 and 90% and 15 populations with frequencies less than 50% (**Table S1**).

The mutant genotype 1534F/S was the most common and was detected in 42 populations, followed by 1534S/S in 36 populations, 1534F/C in 25 populations, 1534F/L in 18 populations, 1534C/C in 15 populations, and 1534S/C in 13 populations. The mutant genotype 1534L/L was found in only three populations: HALY, GXFCG, and GDSZ. The GXNN, GXQZ, GDSZ, and FJHL populations carried the highest number of different mutant genotypes with six, followed by GXFCG, GXBS, CQQJ, CQYB, CQWZ, CQDJ, GDFS, GDGZ, YNMS, and FJXA with five. In these 11 provinces, only Guangxi province had all seven mutant genotypes in the 1534 codon. The highest frequency of mutant genotypes was 92.61% in Chongqing and the lowest was 26.07% in Henan. Besides Henan, there were two provinces, Hubei (48.61%) and Shandong (33.31%), with the mutant frequency less than 50%. The genotypes of F/F, F/S, and S/S were found in all 11 provinces. L/L was found only in three provinces, Henan, Guangxi, and Guangdong. The heterozygote S/C was detected in 10 provinces; the provinces where it was not found were Henan and Zhejiang (**Table S2**).

### Frequency and distribution of triple−locus genotype combinations in the *Ae. albopictus VGSC* gene

From a total of 2241 individuals, we found 31 different triple-locus genotype combinations in *Ae. albopictus* (**Table 2**; **Table S3**). The number of triple-locus genotype combinations observed at each sampling site ranged from 1 (HNZZ) to 14 (HNHY and HNLD). Type 1 (wild type V/V+I/I+F/F), Type 2 (single mutation at locus 1534, V/V+I/I+F/S) and type 5 (V/V+I/I+S/S) were the most common and were found in 37 populations. In addition, seven combinations: type 9 (V/V+I/I+S/L), type 19 (V/G+I/I+L/L), type 23 (G/G+I/I+S/S), type 24 (G/G+I/I+C/C), type 28 (V/V+I/T+S/S), type 30 (V/G+I/T+F/S), and type 31 (V/G+I/T+S/S) were uniquely present in GDGZ, GDSZ, HNHH, GXQZ, HNHY, HBHZ, and HNLD, respectively. Type 1 was the triple-locus wild homozygote and occurred at high frequencies in GDMM (92.50%), HAJZ (91.07%), and GXFCG (71.43%), but at low frequencies in GXBS, HNHH, HNXT, CQQJ, CQWZ, GDFS, GDJM, and YNLC (less than 10%) (**Table 2**, **Table S3**).

**Table 2:** Frequency distribution of triple-locus genotype combinations of *Ae. albopictus* in 11 provinces of China.

Type	Alleles	Henan	Guangxi	Hunan	Chongqing	Hubei	Guangdong	Shandong	Yunnan	Jiangxi	Zhejiang	Fujian
1	V/V+I/I+F/F	32.68	30.71	6.99	0.78	24.51	21.52	30.51	26.06	13.01	15.63	20.47
2	V/V+I/I+F/S	10.12	16.10	13.54	0.39	7.91	24.47	2.26	12.68	17.07	17.97	18.71
3	V/V+I/I+F/C		5.24		7.00	9.49	0.84	11.30				9.36
4	V/V+I/I+F/L	5.84	1.12	2.18			5.06	3.39	0.70			0.58
5	V/V+I/I+S/S	2.72	24.34	44.10	3.11	3.95	32.91	0.56	20.42	21.14	56.25	14.04
6	V/V+I/I+C/C		3.37		29.96			2.26	2.11			3.51
7	V/V+I/I+L/L	0.39	0.37				0.42					
8	V/V+I/I+S/C		8.99	0.44	30.35	3.56	2.53	0.56	2.82			9.94
9	V/V+I/I+S/L						0.42					
10	V/G+I/I+F/F	16.73	1.12	5.24	4.67	9.88	1.69	15.82	3.52	8.13	1.56	11.70
11	G/G+I/I+F/F	4.67	1.12	1.31	1.95	7.11	0.84	1.69	2.82	1.63		0.58
12	V/V+I/T+F/F	12.45		3.49		5.53	0.42	16.95	7.04	8.94	0.78	1.17
13	V/V+T/T+F/F	1.95						1.13	2.82	1.63		
14	V/G+I/I+F/S	1.17	3.75	12.66	5.06	14.62	5.06	1.69	11.27	17.89	6.25	7.60
15	V/G+I/I+F/C				10.12	2.77	0.42	6.21	0.70			
16	V/G+I/I+F/L	3.50	0.37	0.87			1.69	0.56				0.58
17	V/G+I/I+S/S	0.39	0.37	3.06	0.39		0.42					0.58
18	V/G+I/I+C/C		1.50		1.56							
19	V/G+I/I+L/L						0.42					
20	V/G+I/I+S/C				0.78							
21	G/G+I/I+F/S			0.87				0.56				
22	G/G+I/I+F/C		1.12			0.79	0.42	0.56				1.17
23	G/G+I/I+S/S			0.44								
24	G/G+I/I+C/C		0.37									
25	V/V+I/T+F/S	0.39		1.75		3.95			6.34	5.69	0.78	
26	V/V+I/T+F/C				0.78	0.79		1.69				
27	V/V+I/T+F/L	1.56		0.44								
28	V/V+I/T+S/S			0.44								
29	V/G+I/T+F/F	5.45		1.31		4.35	0.42	1.69	0.70	3.25		
30	V/G+I/T+F/S					0.40						
31	V/G+I/T+S/S			0.44								

Type 1 indicates no mutation in three codons; Type 2 to 13 indicates mutation in one codon; Type 14 to 29 indicates mutation in two codons; Type 30 to 31 means mutation in three codons.

The presence of single mutation was observed at loci 1016, 1532, and 1534 (types 2 to 13). The combinations (types 2 and 10) heterozygous at one insecticide resistance related locus (either 1016 or 1534) were widely distributed in 37 and 36 populations, respectively. Combinations (types 14 to 29) were found at two of the three loci (i.e., 1016 + 1532, 1016 + 1534, or 1532 + 1534); type 14 was widely distributed in 35 populations. Single mutant homozygotes 1016 G/G (type 11), 1532 T/T (type 13), 1534 S/S (type 5), 1534 C/C (type 6), and 1534 L/L (type 7) were present in 26, 7, 37, 14, and 3 populations, respectively. In this study, we also found triple-locus mutant individuals. The combination (type 30) that was heterozygous at three loci (V/G + I/T +F/S) were only detected in HBHZ and the combination (type 31) that was heterozygous at two of three loci and homozygotes at one of three loci (V/G +I/T +S/S) were only detected in HNLD.

### The comparison between dengue and non-dengue epidemic areas

Based on whether there was a local outbreak of dengue fever prior to 2019, the sampling sites were divided into two groups: dengue epidemic areas (DEAs) (1) and non-dengue epidemic areas (NDEAs) (2). The three sites in Yunnan were all NDEAs and three sites in Zhejiang were all DEAs. In total, there were 24 DEAs and 25 NDEAs. We compared the *kdr* mutation rates between all DEAs and NDEAs, and the results showed there was no significant difference in the 1016 and 1532 mutation rates, but there was a significant difference in the 1534 mutation rate (*χ^2^* = 10.357, *P* < 0.001). The *kdr* gene mutation rates between DEAs and NDEAs in each province except for Zhejiang and Yunnan. The results showed that there was a significant difference (*P* < 0.05) in the 1016, 1532, and 1534 mutation rate between the two groups only in Henan and Hunan province. In Guangdong, there was a significant difference in the 1016 (*χ^2^* = 5.0381, *P* = 0.0248) and 1534 (*χ^2^* = 71.795, *P* < 0.001) mutation rate between the two groups. In Guangxi and Chongqing provinces, there was a significant difference in the 1016 mutation rate between the two groups. In Jiangxi, there was a significant difference in the 1532 (*χ^2^* = 5.6412, *P* = 0.01754) mutation rate between the two groups. In Shandong and Fujian provinces, there was a significant difference in the 1534 mutation rate between the two groups (**Table 3**).

**Table 3:** Comparison of mutation rates between dengue epidemic areas and non-dengue epidemic areas.

Group	1016					1532					1534			
	Wild	Mutant	*χ^2^*	*P*		Wild	Mutant	*χ^2^*	*P*		Wild	Mutant	*χ^2^*	*P*
Total 1	1856	290	0.166	0.683		2036	110	0.509	0.476		1082	1064	10.357	0.001
Total 2	2030	306				2227	109				1290	1046		
Henan1	221	83	39.001	<0.001		243	61	45.913	<0.001		268	36	4.5621	0.03269
Henan2	199	11				210	0				170	40		
Guangxi1	277	9	8.676	0.0032		286	0	—	1.000		143	143	2.2389	0.1346
Guangxi2	224	24				248	0				107	141		
Hunan1	148	34	3.910	0.0479		164	18	25.854	<0.001		102	80	60.475	<0.001
Hunan2	244	32				276	0				56	220		
Chongqing1	200	42	6.119	0.0133		242	0	—	0.5007		53	189	2.0469	0.1525
Chongqing2	246	26				270	2				45	227		
Hubei1	125	33	1.514	0.218		151	7	2.525	0.112		109	49	0.78891	0.3744
Hubei2	256	92				317	31				255	93		
Guangdong1	288	26	5.038	0.0248		312	2	—	0.5518		94	220	71.795	<0.001
Guangdong2	156	4				160	0				114	46		
Shandong1	70	8	1.820	0.1773		68	10	0.077	0.781		76	2	15.721	<0.001
Shandong2	228	48				246	30				212	64		
Jiangxi1	131	21	1.822	0.1771		142	10	5.641	0.01754		79	73	3.4443	0.06347
Jiangxi2	74	20				78	16				61	33		
Fujian1	150	24	0.493	0.4824		174	0	—	0.2406		78	96	8.6698	0.003235
Fujian2	150	18				166	2				103	65		

In the first column, 1 refers dengue epidemic areas; 2 refers non-dengue epidemic areas; —, no chi-square value was obtained and fisher’s exact test was used.

### Correlation analysis between *VGSC* gene mutation rates and meteorological factors

**Figure 4:**
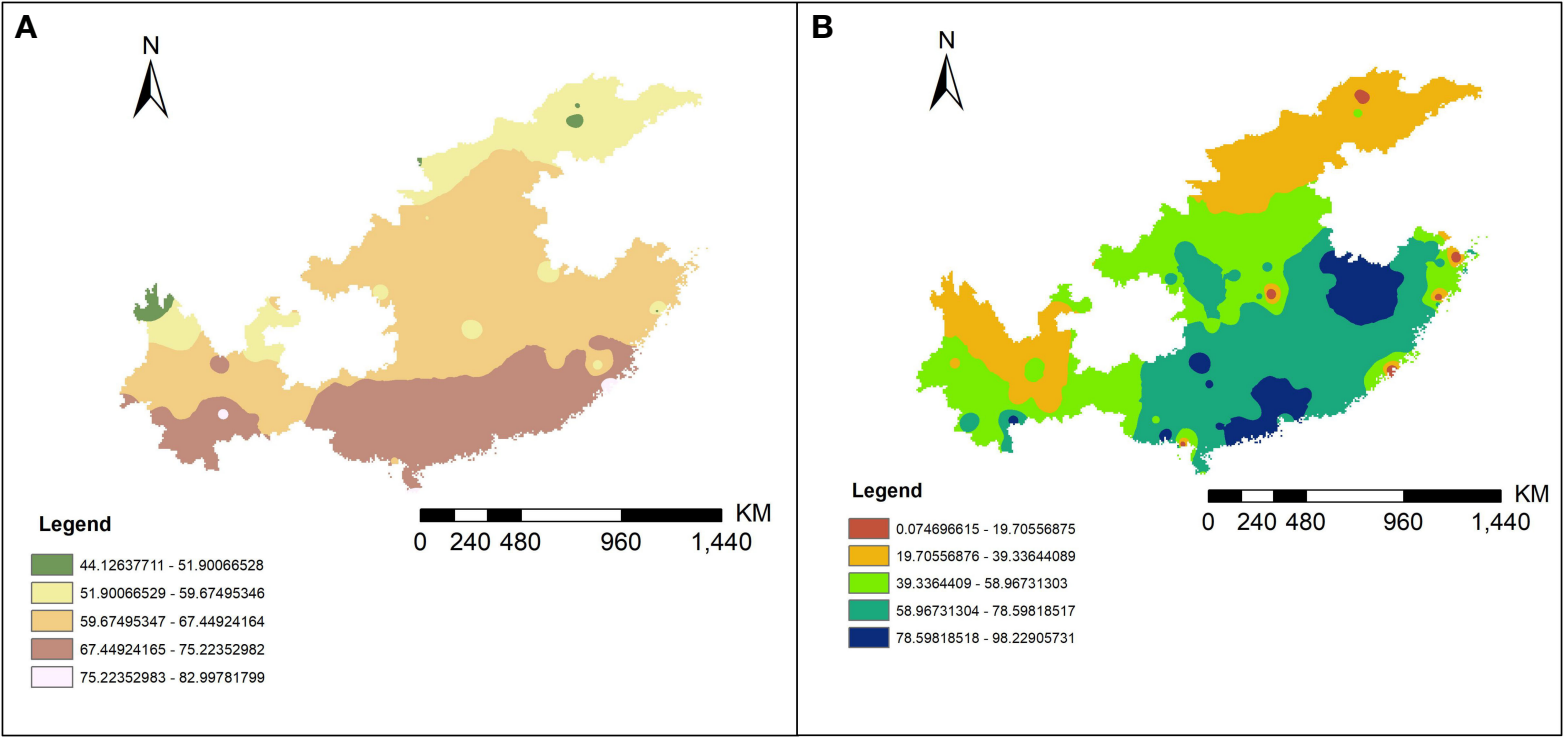
The predictive results map of the AAT **(A)** and AAR **(B)** in 11 provinces of China from 2011 to 2020 by IDW interpolation method.

**Figure 5:**
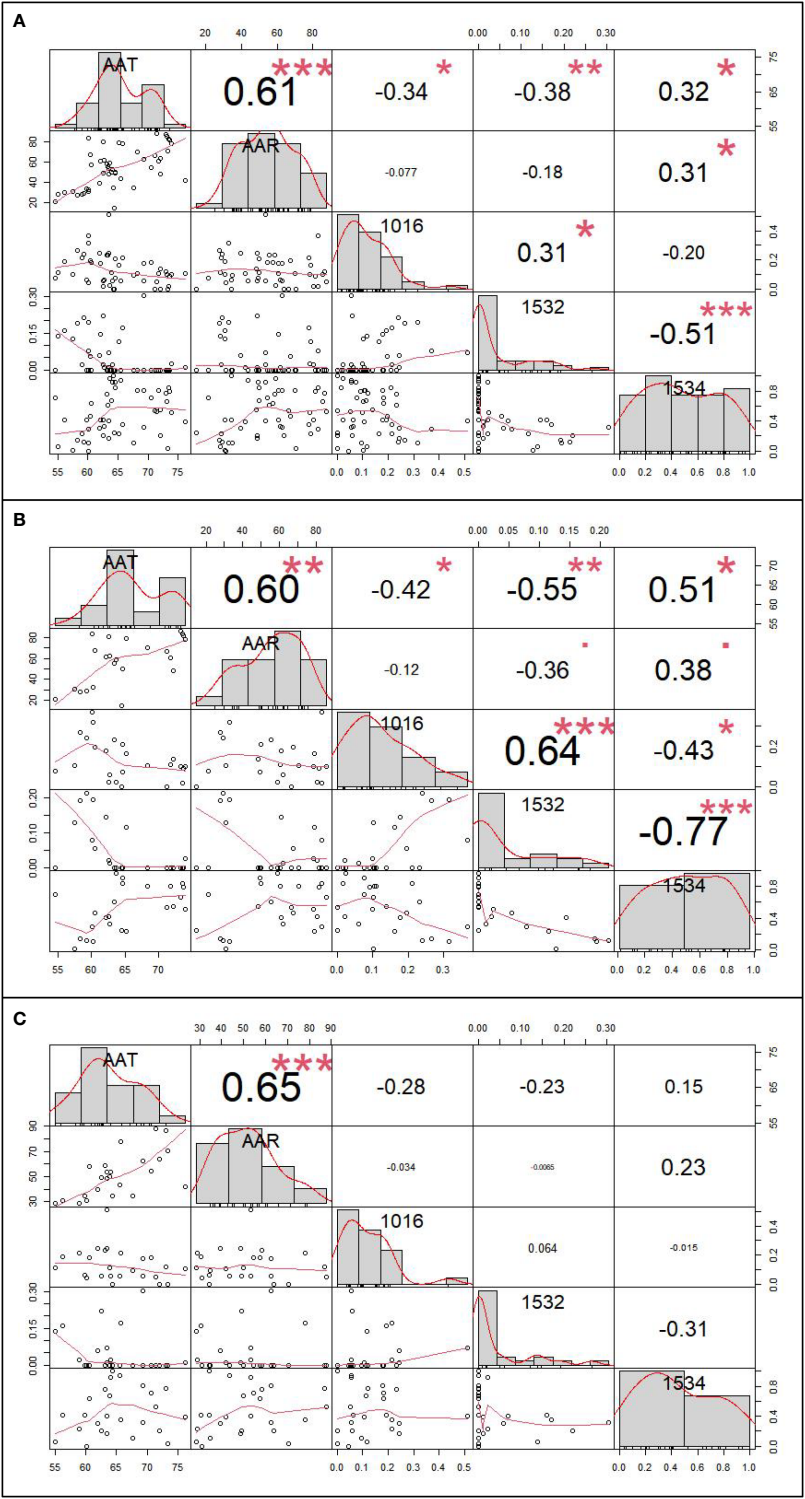
The correlation analysis result between *kdr* mutation rates and meteorological factor. **(A)** The correlation analysis result among 49 sample collection sites; **(B)** The correlation analysis among 24 sample collection sites of dengue epidemic areas; **(C)** The correlation analysis among 25 sample collection sites of non-dengue epidemic areas.* refer to *P*<0.05, ** refer to *P*<0.01, and *** refer to *P*<0.001.

The inverse distance weighted (IDW) results are shown in **Figure 4** and the extraction data of collection sites are in the **Table S4**. By correlation analysis we know that the 1016 and 1532 codon mutation rates were significantly negatively related to annual average temperature (AAT) (Pearson correlation: r (49) = −0.34, *P* < 0.05; r (49) = −0.38, *P* < 0.01) and the 1534 codon mutant rate was significantly positively related to AAT and average annual rainfall (AAR) (Pearson correlation: r (49) = 0.32, *P* < 0.05; r (49) = 0.31, *P* < 0.05). The 1532 codon mutation rate was significantly positively related to the 1016 codon mutant rate (Pearson correlation: r (49) = 0.31, *P* < 0.05) and significantly negatively related to the 1534 codon mutant rate (Pearson correlation: r (49) = −0.51, *P* < 0.001). There was no significant relationship between the 1016 codon and 1534 codon mutation rates. Dividing the sites into two categories, including DEAs and NDEAs, another correlation analysis was conducted. The results showed in DEAs, there were still significant negative relationship between AAT and the 1016 codon mutation rate and 1532 and 1534 codon mutation rates (Pearson correlation: r (49) = −0.42, *P* < 0.05; r (49) =−0.55, *P* < 0.01; r (49) = 0.51, *P* < 0.05) but no significant relationship with AAR. The 1532 codon mutant rate was significantly positively related to 1016 codon mutant rate (Pearson correlation: r (49) = 0.64, *P* < 0.001) and significantly negatively related to the 1534 codon mutation rate (Pearson correlation: r (49) = −0.77, *P* < 0.001). The correlation index was increased and there was a significant relationship between the 1016 and 1534 codon mutation rates (Pearson correlation: r (49) = −0.43, *P* < 0.05). In NDEAs, the results showed there was no significant relationship between these variables (**Figure 5**).

### Global autocorrelation and local autocorrelation analysis

Through global spatial autocorrelation analysis, it was found that mutation rates of loci 1016, 1532, and 1534 were positive spatial correlation. Moran’s *I* indexes were 0.180 (*Z* = 2.146, *P* = 0.032), 0.177 (*Z*= 2.113, *P* = 0.035) and 0.475 (*Z* = 5.14, *P* < 0.001), respectively. In addition, the spatial distribution pattern showed it was clustered.

**Figure 6:**
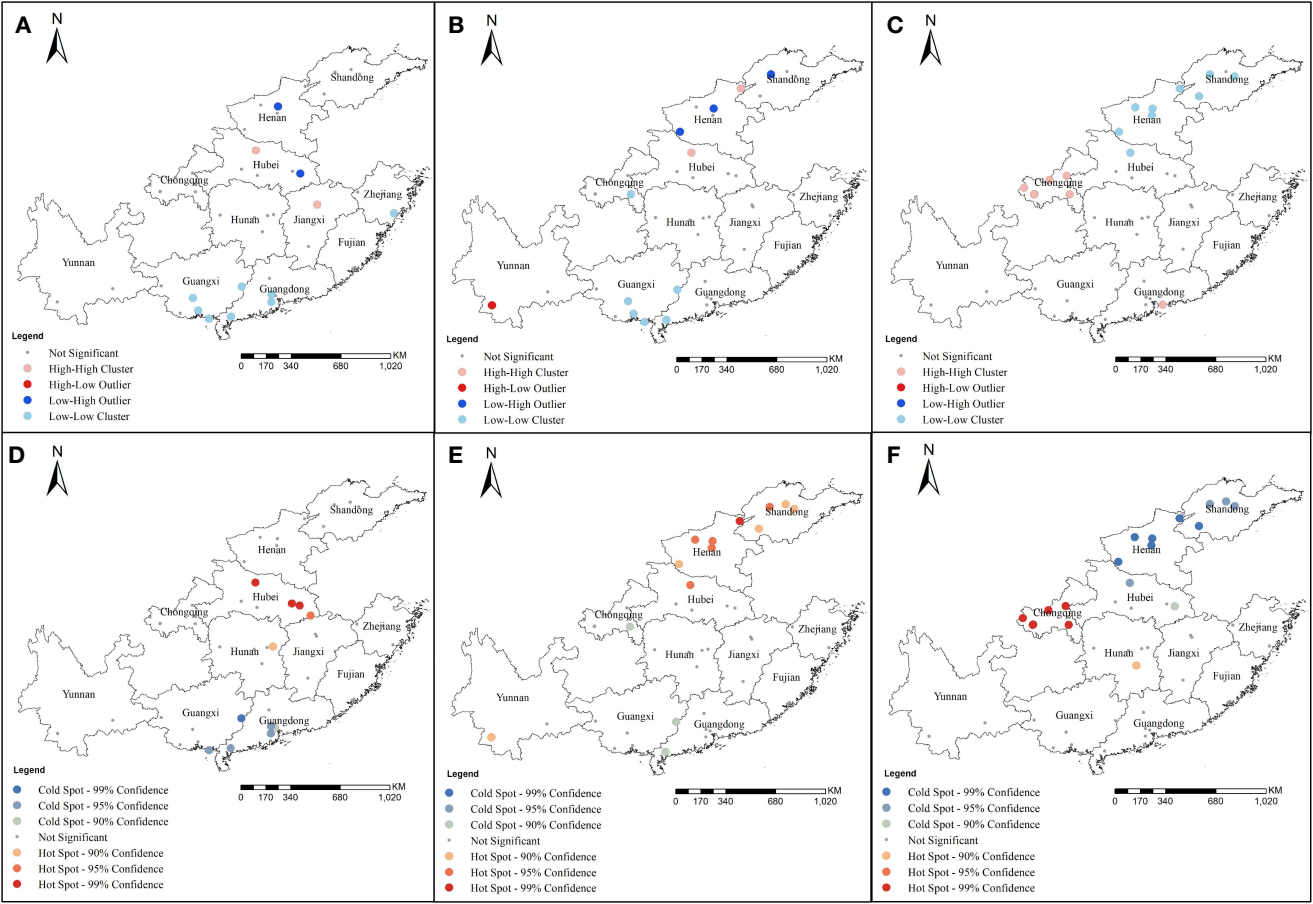
Local spatial autocorrelation for mutation rates of different codons of *Ae. albopictus VGSC* gene in 2020 at 49 sample collection sites from 11 provinces. **(A)** and **(D)** were the results of the Cluster and outlier analysis (Anselin Local Morans *I*) and Hot Spot analysis (Getis-Ord Gi*) at codon 1016 of *Ae. albopictus VGSC* gene; **(B)** and **(E)** were the results of the Cluster and outlier analysis (Anselin Local Morans *I*) and Hot Spot analysis (Getis-Ord Gi*) at codon 1532 of *Ae. albopictus VGSC* gene; **(C)** and **(F)** were the results of the Cluster and outlier analysis (Anselin Local Morans *I*) and Hot Spot anal-ysis (Getis-Ord Gi*) at codon 1534 of *Ae. albopictus VGSC* gene.

Local spatial autocorrelation analysis showed the Anselin local Moran’s *I* analysis of the 1016 codon mutant rate showed high−high clusters in some areas of Hubei and Jiangxi. However, low−low clusters were detected in some areas of Guangxi, Guangdong, and Zhejiang (**Figure 6A**). At locus 1532, high−high clusters were detected in Henan and Hubei and low−low clusters were detected in Guangxi and Guangdong (**Figure 6B**). At locus 1534, high−high clusters were mainly at Chongqing and Guangdong and low−low clusters were at Henan, Hubei, and Shandong (**Figure 6C**). Getis-Ord Gi* analysis showed that the mutation rates at 1016 had formed hot spots at some sites in Hubei and Hunan, and cold spots were mainly located in Guangdong and Guangxi (**Figure 6D**). At locus 1532, hot spots were mainly concentrated in Hubei, Henan, Shandong, and Yunnan, whereas cold spots were mainly concentrated in Guangxi and Guangdong (**Figure 6E**). At locus 1534, hot spots were mainly concentrated in Chongqing and Hunan, whereas cold spots were mainly concentrated in Henan, Shandong, and Hubei (**Figure 6F**).

## Discussion

In 2019, there were 15,187 locally transmitted dengue cases in 13 provinces of mainland China, making it the worst year of the epidemic during the period 2005–2018, except for 2014 (Yue et al., 2021). Overseas imported cases of dengue fever were reported in all provinces of mainland China until 2019, except for the Tibet Autonomous Region (Lin et al., 2020). In the absence of an effective vaccine for dengue fever, vector control is crucial. We explored whether resistance of dengue vector *Ae. albopictus* affects the local outbreaks of dengue fever. In this study, we selected sample collection sites from 11 provinces, excluding Hainan and Sichuan provinces. The sites included dengue epidemic areas (DEAs) (i.e., there was a local outbreak of dengue fever) and non-dengue epidemic areas (NDEAs). *Ae. albopictus* is the main vector of dengue fever in China. Therefore, it is vital to study *Ae. albopictus* resistance mechanisms to control the dengue vector effectively. There was a long history of controlling mosquitoes using pyrethroid insecticides in China, which has directly or indirectly brought heavy selection pressure on vector populations resulting in evolved resistance (Cui et al., 2006).

In this study, we sampled the highest number of field populations to date for *Ae. albopictus kdr* mutations research, including 49 populations from 11 provinces and three locus mutations at loci 1016, 1532, and 1534 of the *VGSC* gene. The results showed that the different types of *kdr* mutation had a wide distribution and complex diversity. Overall, we found six alleles and eight genotypes at the 1534 codon which had the most mutation types shown to date. As shown in previous studies in China (Chen et al., 2021; Wu et al., 2021), 1534TCC/S was the predominant mutation detected in our study which was found at HNZZ with a frequency of 100%. In some populations, such as HNZZ, JXGZ, ZJYW, there were no wild-type homozygous mosquitoes at codon 1534. Of the three loci, mutations at the 1532 codon occurred at the fewest populations which was in 22 populations. For loci 1016 and 1534, mutations occurred in 44 and 48 populations, respectively. Therefore, this phenomenon needs to be closely monitored as the *kdr* mutations have occurred at high frequencies in China. Compared with the 1016 and 1534 codons, the mutation rate of the 1532 codon was very low with the highest frequency of 30.43% in the YNLC population. Currently, there is still controversy regarding the correlation between 1532T mutation and pyrethroid resistance (Gao et al., 2018). We also found that combined mutation at codons 1532 and 1534 c was common, consistent with other studies (Zhou et al., 2019; Chen et al., 2021; Wu et al., 2021).

In total, we found 31 different combinations of alleles at the 1016, 1532, and 1534 codons. The wild-type at all three loci and single mutation at codon 1534S were the most common combinations. The least common combination had mutations at each of the three loci.

As vector control is the only effective measure for controlling dengue, insecticide use is very important. Thus, we assumed in dengue fever epidemic areas, the selection pressure would be more severe. We compared the difference of the mutation rates at three loci between dengue epidemic areas and non-dengue epidemic areas in 9 provinces. The results showed no obvious rule. In Henan province, the mutation rates at the three loci were higher in dengue epidemic areas, which explains the widespread use of pyrethroid insecticides to decrease mosquito density and control dengue fever in recent years in these areas. In other provinces, such as Hunan, Hubei, and Shandong, we observed that the 1534 mutation rate was significantly higher in non-dengue fever epidemic areas. This may be due to different provinces using various vector control strategies (Bouzid et al., 2016; Deming et al., 2016). In 1978, dengue fever re-emerged in Guangdong province of mainland China and impacted many provinces, such as Hainan, Guangxi, Fujian and Yunnan during 1980s (Lai et al., 2015). In 2014, there was an explosive outbreak of dengue fever in Guangdong; the number of reported cases was more than three times of the total number in the past 20 years (Zhu et al., 2019). Guangdong province is located on the coast and is one the most economically-developed areas in China. Many imported cases were brought from neighboring countries each year (Zhang et al., 2020). Therefore, it is crucial to study dengue vector resistance to prevent and control dengue outbreaks in advance. In our study, there were six sample collection sites from Guangdong comprising four DEAs and two NDEAs. The results showed that the *kdr* mutation rate was already very high, especially in GDGZ (Guangzhou city) with 80.68% mutation rate at the 1534 codon and GDJM (Jiangmen city) with 84.48% mutation rate at the 1534 codon. In Zhejiang province, we selected three collection sites, which were all DEAs. The frequency of 1534 codon mutations is almost consistent with the study by Wu et al. (Wu et al., 2021) in 2019 involving three populations. The alleles at the 1534 codon only included TTC(F) and TCC(S); this indicates that the populations are highly stable, there is not considerable gene exchange with other populations, and the samples in our study were highly representative of the populations.

Chen et al. reported (Chen et al., 2021) a significant correlation between *kdr* mutations and annual average temperature (AAT). In our study, we also analyzed the relation between the three locus mutations and meteorological factors. In addition to AAT, average annual rainfall (AAR) also affects the mosquito’s breeding and density. Therefore, we wanted to determine if it also affects the *kdr* mutation frequency of *Ae. albopictus*. Our extensive study observed 49 populations from different areas of China to further explore the correlation. Our results showed that AAT was significantly positively related to 1534 mutation rate but negatively related to 1532 mutation rate. In addition, we also showed that AAT was significantly negatively related to 1016 mutation rate similar to the 1532 codon mutation rate. However, for AAR, there was only a positive relationship with the 1534 mutation rate. We also found that the 1532 mutation rate was positively related to 1016 but negatively related to 1534. In order to verify the correlation, we separated the areas into two categories according to dengue fever local outbreak. In the dengue fever epidemic areas, there was a stronger relationship between AAT and *kdr* mutation, but the relationship disappeared in non-dengue epidemic areas. This could be explained by meteorological factors affecting the occurrence of dengue fever (Shen et al., 2015; Choi et al., 2016). Gao et al. (Gao et al., 2018) and Wu et al. (Wu et al., 2021)reported that I1532T is negatively correlated with the pyrethroid insecticide resistance phenotype but F1534S is positively correlated with pyrethroid insecticide resistance phenotype. Our study considered the correlation of three *kdr* loci and the negative correlation between 1532 and 1534, which is consistent with previous studies (Gao et al., 2018; Wu et al., 2021). No significant correlation was detected between the mutations at 1016 and 1534 which could be attributed to the distance between these two codons in the *VGSC* gene. We assume that 1016 and 1534 have different mechanisms in affecting insecticide resistance, possibly producing resistance to different types of pyrethroid insecticides.

This study is the first to use spatial autocorrelation analysis to analyze the spatial aggregation of *VGSC* gene mutations in *Ae. albopictus* from different provinces in China. Analysis of global spatial autocorrelation results showed that the three common mutation loci of *Ae. albopictus VGSC* gene, 1016, 1532, and 1534, showed spatial aggregation and positive spatial correlation. These results suggest that mutations in the *VGSC* gene of *Ae. albopictus* between adjacent regions may affect the surrounding populations. Furthermore, local autocorrelation analysis showed that the mutation rate was consistent at loci 1016 and 1532, and the mutation rate was low in high latitude regions such as Guangdong and Guangxi. Mutation rates were higher in low latitude areas, such as Shandong, Henan, and Hubei, which was consistent with the positive correlation between them in the previous correlation analysis. However, at locus 1534, the cold spot areas were exactly in the hot spot areas of loci 1016 and 1532, which showed a negative correlation. All sampling sites in Chongqing showed high-high clusters, indicating a high mutation rate at codon 1534 in the whole province. The varying characteristics of different provinces may be related to the local mosquito density and the selection and use of insecticides, which requires further investigation.

There are a few limitations in our study. First, we collected the samples by different methods for adults and larvae, which may influence the comparability of the results. Second, the relationships among *Ae. albopictus* density, bioassay results and the local level of insecticides usage needs to be further explored.

This study described the distribution of *kdr* gene mutation in the dengue vector *Ae. albopictus* in China. It looks at the highest number of field populations for *Ae. albopictus kdr* mutations to date. The association between insecticide resistance of *Ae. albopictus* and dengue fever outbreak was explored. Correlation analysis among three loci mutation rates at 1016, 1532 and 1534 provided a new method to study molecular mechanism of insecticide resistance. The spatial aggregation of *VGSC* gene mutations showed the gene exchange and similarity of insecticide usage in the adjacent areas. In the future, we will pay more attention to high mutation rate areas like Chongqing and Guangdong provinces and adjust insecticide use plan to delay resistance development.

## Data availability statement

The data presented in the study are deposited in the NCBI repository, accession number OP595565-OP595600, OP609959-OP610054, OP765517-OP765559, OP781335-OP781414, OP792043-OP792755, OP807490-OP807752, OP830531-OP830807, OP853152-OP853408, OP884691-OP885186, OP940152-OP941127, OP948914-OP950158.

## Author contributions

CZ performed data analysis and original draft preparation. XZ conducted samples detection and experimental analysis. CX conducted statistical analysis. XLu and WL participated in laboratory detection work. XLi and HW supervised the writing of the manuscript. XS and JW devoted to laboratory quality control. QL reviewed final draft and provided revision advice. FM contributed to project administration and supervision. All authors have read and agreed to the published version of the manuscript.

## Funding

This research was funded by the National Science and Technology Major Project of China (No. 2018ZX10101002-002) and Special project of Chinese Center for Disease Control and Prevention: “Special investigation on dengue vectors”.

## Acknowledgments

We really appreciate all Centers for Disease Prevention and Control (CDCs) from Henan, Guangxi, Hunan, Chongqing, Hubei, Guangdong, Shandong, Yunnan, Jiangxi, Zhejiang, and Fujian to provide mosquitoes samples.

## Conflict of interest

The authors declare that the research was conducted in the absence of any commercial or financial relationships that could be construed as a potential conflict of interest.

## Publisher’s note

All claims expressed in this article are solely those of the authors and do not necessarily represent those of their affiliated organizations, or those of the publisher, the editors and the reviewers. Any product that may be evaluated in this article, or claim that may be made by its manufacturer, is not guaranteed or endorsed by the publisher.

## Supplementary material

Supplementary Table 1 Alleles and frequency of *kdr* mutation of *Aedes albopictus* in field populations of this study. (XLSX);

Supplementary Table 2 Genotypes and frequency of *kdr* mutation of *Aedes albopictus* in field populations of this study. (XLSX);

Supplementary Table 3 Frequency distribution of triple-locus genotype combinations in 49 *Aedes albopictus* field populations of this study. (XLSX);

Supplementary Table 4 The annual average temperature (AAT) and the average annual rainfall (AAR) of sample collection sites extracted from inverse distance weighted (IDW) interpolation results. (XLSX).
